# Local injection of botulinum toxin type A in the treatment of thalamic ataxia syndrome: A case report

**DOI:** 10.1097/MD.0000000000043136

**Published:** 2025-07-11

**Authors:** Tingting Zhang, Yike Zhang, Jiangbo Xie, Manli Zhao, Peichun Li

**Affiliations:** a Weifang Hospital of Traditional Chinese Medicine, Shandong Second Medical University, Weifang, China; b Shandong University of Traditional Chinese Medicine, Jinan, China.

**Keywords:** botulinum toxin type A, case report, rehabilitation, thalamic ataxia syndrome, thalamus

## Abstract

**Rationale:**

Thalamic ataxia syndrome is a rare but *severely disabling* condition resulting from thalamic lesions, *characterized by persistent motor incoordination that frequently fails to respond* to conventional rehabilitation therapies. *Given the lack of effective treatment options and the profound impact on patients’ quality of life, this study aimed to evaluate* the efficacy of local botulinum toxin type A (BoNT-A) injections *as a potential breakthrough treatment* for improving motor control and daily living activities in patients with thalamic ataxia. *This investigation represents the first clinical application of BoNT-A specifically targeting thalamic ataxia, offering new hope for treatment-resistant cases.*

**Patient concerns:**

A 59-year-old female presented with a 6-month history of debilitating left limb movement disorder, including inability to lift the left upper limb, grasp objects, or perform essential daily activities such as eating and carrying a cup. Despite 5 months of *intensive* rehabilitation, her symptoms showed minimal improvement, *leaving her functionally dependent.*

**Diagnosis:**

The patient was diagnosed with thalamic ataxia syndrome secondary to right thalamic hemorrhage, confirmed by cranial CT and MRI.

**Interventions:**

The patient received precisely targeted local BoNT-A injections (total 130U) into the affected upper limb muscles (deltoid, biceps brachii, flexor carpi radialis, flexor carpi ulnaris, pronator teres, extensor carpi radialis, and flexor digitorum superficialis) under dual ultrasound and electromyography guidance, ensuring optimal muscle selection and dosing accuracy.

**Outcomes:**

Three weeks postinjection, the International Cooperative Ataxia Rating Scale score decreased by 8 points (from 24 to 16), with the most significant improvement in dynamic function (upper limb ataxia subscores reduced from 18 to 10). The Fugl-Meyer Assessment Upper Extremity Scale increased by 6 points (from 52 to 58), particularly in hand coordination and speed tasks. The modified Barthel index improved by 9 points (from 85 to 94), enabling independent performance of previously difficult activities like self-feeding and cup handling.

**Lessons:**

Local BoNT-A injections *represent a novel and potentially transformative* treatment for thalamic ataxia syndrome, particularly in cases refractory to conventional therapies. These findings challenge current rehabilitation paradigms and highlight the need for further studies with larger cohorts to validate and expand upon these findings.

## 1. Introduction

Thalamic ataxia syndrome is a relatively rare clinical syndrome caused by thalamic lesions, characterized by contralateral “cerebellar” ataxia, with or without hemiparesis, and typically without or only transient sensory deficits.^[1]^ The ataxia associated with this condition poses significant challenges for rehabilitation. Currently, commonly employed rehabilitation strategies for ataxia include proprioceptive neuromuscular facilitation techniques, coordination training, balance training, and transcranial magnetic stimulation. However, these approaches often require prolonged treatment courses and yield limited therapeutic efficacy. Based on the clinical features of ataxia, we utilized localized injections of botulinum toxin type A (BoNT-A) into the relevant responsible muscles to treat a patient with this condition. The case is reported as follows.

## 2. Case presentation

A 59-year-old female patient came to our hospital for treatment of left limb movement disorder for more than 6 months. The patient had a history of hypertension, no systematic oral antihypertensive drugs, a blood pressure of up to 185/110 mm Hg, and no history of type 2 diabetes, cardiovascular disease, or peripheral vascular disease.

The patient had a history of smoking and drinking but had quit smoking for 5 years. The patient’s family history was unremarkable.

**Figure 1.**
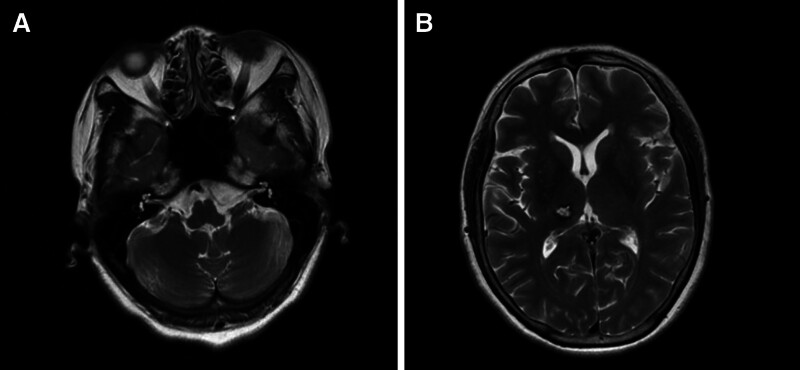
(A, B) Cerebral MRI showed hemorrhages in the right thalamus with hemosiderosis.

Six months before admission, the patient developed left limb movement disorder without obvious causes or inducement, which was manifested as inability to lift the left upper limb, inability to grasp the left hand, instability in walking, nausea and vomiting, and the vomit was stomach contents. At the time of onset, she did not experience incontinence or limb convulsions, and her family members rushed her to the local hospital for treatment, where cranial CT showed bleeding in the right thalamus. After hemostasis, blood pressure control, dehydration, cranial pressure reduction, nutritional nerve, and symptomatic treatment, the patient’s vital signs gradually stabilized, the left limb could move autonomously and walk independently, but the left limb movement was not coordinated, the upper limb symptoms were more severe, and the lower limb symptoms were less severe. 5 months before admission, the patient underwent systematic rehabilitation treatment in several hospitals in the city in order to improve the coordination dysfunction of the left limb. However, the improvement effect of the coordination of the left limb was not obvious, and the patient still could not carry a cup by himself, eat with a spoon in his left hand and other daily life activities. The vital signs examination in this admission were as follows: body temperature 36.5℃, heart rate 70 times/min, breathing 18 times/min, blood pressure 125/80 mm Hg. The patient was alert and oriented, clear speech, no dysarthria. Double pupils are large and round, with a diameter of about 3mm, sensitive to light reflection, normal anisotropic activity, and no nystagmus. Right limb strength level 5, left limb strength level 5. The left finger nose test was not stable and the left heel knee tibial test was not good. Ataxia is heavier on the upper limbs than on the lower limbs. Intentional tremor in the left upper limb. The pain and temperature of the left side of the body decreased, the motion, position and vibration senses were normal, and Romberg sign was negative. Holden Walking function rating 4. Left side pathological signs were negative, other physical examination showed no abnormal findings. Laboratory examination showed no abnormality in blood routine, biochemistry, CRP, thyroid function, and surgical synthesis. Cardiopulmonary examination and cervical vascular ultrasound showed no abnormal findings. Cerebral MRI showed hemorrhages in the right thalamus with hemosiderosis (Fig. 1A, B). The patient was diagnosed with thalamic hemorrhage, which later caused thalamic ataxia.

After hospitalization, the patient was given 30 mg nifedipine controlled release tablet every morning to control blood pressure. The patient had been treated with rehabilitation and acupuncture for 5 months in the past, but the treatment effect of ataxia was not good, and rehabilitation and acupuncture treatment were not performed in this diagnosis and treatment. Because the patient’s left lower limb ataxia was relatively mild and did not affect the patient’s daily walking, the lower limb BoNT-A local injection treatment was not performed. The patient suffered from severe ataxia in the left upper limb and had difficulty completing daily living activities requiring the participation of the left upper limb. Under the guidance of surface electromyography combined with ultrasound, A certain dose of BoNT-A was injected locally into the relevant responsible muscles of the left upper limb (Table 1). Dosage distribution: deltoid 30U, biceps brachii 20U, flexor carpi radialis 20U, flexor carpi ulnaris 15U, pronator teres 15U, extensor carpi radialis 15U, and flexor digitorum superficialis 15U (total 130U).

**Table 1:** Local injection site and dosage of botulinum toxin type A were performed on the relevant responsible muscle of the left upper limb.

Muscles injected	Deltoid	Bicep	FCR	FCU	Pronator teres	ED	FDS
Dose injected	15 U	30 U	15 U	15 U	15 U	10 U	30 U

In terms of rehabilitation assessment methods, the assessment was carried out by the same professional before injection and 3 weeks after injection, after the patient had a full rest, and the surrounding environment was required to be quiet and without interference, and the family members or escorts were around to take care of the patient to eliminate the tension. The International Cooperative Ataxia Rating Scale (ICARS)^[2]^ was used to assess the patient’s ataxia. ICARS has a total score of 100, based on all 19 items. The scale included postural and gait disorders (34 points), dynamic function (ataxia, 52 points), speech disorders (8 points), and eye movement disorders (6 points). Fugl-Meyer Assessment (FMA) Upper Extremity Scale. There are 33 items in the upper limb, including body reflexes, cooperative movements of flexor and extensor muscles, activities with cooperative movements, activities out of cooperative movements, wrist stability, hand grip strength, hand flexor and extension, finger and nose coordination and speed. Each item is divided into 3 levels; A score of 0 means that it cannot be done, a score of 1 means that it can be done partially, and a score of 2 means that it is fully completed. A perfect score of 66. The modified Barthel index^[3]^ was used to assess the ability to perform activities of daily living.

Compared with before injection, the ICARS score of the patient decreased by 8 points (from 24 to 16, with kinetic function subscores improving from 18 to 10) and the dynamic function (upper limb ataxia) score was significantly improved after 3 weeks of injection. Because the patient had no obvious walking dysfunction, speech dysfunction, and eye movement dysfunction, there was no significant change in the scores of abnormal posture and gait, speech disorder, and eye movement. FMA improved the upper limb by 6 points (from 52 to 58), mainly manifested in the improvement of hand coordination and speed (three coordination tasks progressed from partial to complete execution). The modified Barthel index increased by 9 points (from 85 to 94), and the total score was close to the full score. After the injection, the basic daily life of the patients could be completed (self-feeding speed improved from 3 to 8 bites/minute; stable cup-holding with 200 mL liquid), and the return to family was truly realized. The upper limb of FMA scale improved by 6 points compared with that before injection, mainly in the improvement of coordination ability and speed (Table 2). Finger-to-nose test showed 40% reduction in tremor amplitude.

**Table 2:** Timeline of clinical assessments.

Assessment period	ICARS	MBI	Fugl-Meyer (Upper limb component)	Key observations
Preinjection	24	85	52	Severe upper limb ataxia, dependency in ADLs
3 Weeks after injection	16 (−8)	94 (+9)	58 (+6)	Independent feeding, stable grip

ADL = activities of daily living, ICARS = International Cooperative Ataxia Rating Scale, MBI = modified Barthel index.

## 3. Discussion

The regulation of cerebellar motor function is critically dependent on the frontal-pontine-cerebellar-thalamic-sensory-motor cortical loop.^[4]^ Cortical information is transmitted to the middle cerebellar peduncle via the pontine nucleus, while motor feedback is relayed to the inferior cerebellar nucleus through the inferior olivary nucleus. Processed cerebellar information is then conveyed to the primary motor cortex via the thalamus and superior cerebellar peduncle. Lesions at any point within this pathway can result in ataxia. In this case, the patient exhibited no significant deficits in deep sensory function, thereby excluding sensory ataxia caused by severe deep sensory impairment. Given the presence of thalamic hemorrhage, ataxia, and superficial sensory deficits, and the absence of cerebellar lesions on cranial MRI, it was concluded that the ataxia was not due to cerebellar pathology but rather to thalamic damage. Thus, the ataxia in this patient likely arose from disruption of the dentate nucleus-thalamic pathway secondary to thalamic hemorrhage.

Currently, common rehabilitation approaches for ataxia include balance training, proprioceptive neuromuscular facilitation techniques, coordination training, and repetitive transcranial magnetic stimulation. However, these methods often require prolonged treatment courses and generally yield limited efficacy, as evidenced by our patient’s 5-month treatment plateau prior to BoNT-A intervention. Naoki et al^[2]^ demonstrated that combining 1 Hz low-frequency repetitive transcranial magnetic stimulation applied to the contralateral cerebral hemisphere with 120 minutes of intensive occupational therapy daily in 7 patients with thalamic ataxia led to improvements in motor function and alleviation of ataxia, as assessed by FMA and ICARS scores.

BoNT-A exerts its effects by acting on cholinergic nerve endings in skeletal muscle, inducing muscle paralysis through the inhibition of acetylcholine release from presynaptic membranes. Additionally, it suppresses glandular secretion via vasoactive intestinal peptide-mediated sympathetic and parasympathetic inhibition.^[5,6]^ Kikuchi et al^[7]^ reported that local injections of BoNT-A in patients with spinal cerebellar ataxia type 1 and cervical dystonia resulted in alleviation of ataxia. They also observed hypermetabolic inhibition in the bilateral chiasmatic nuclei and primary sensorimotor cortex following treatment, suggesting that long-term relief might be achievable with repeated injections. Similarly, Nicki et al^[8]^ found that local injections of BoNT-A into upper limb-related muscle groups significantly improved tremor in patients with idiopathic tremor, Parkinson disease tremor, and cerebellar tremor. Previous studies^[9,10]^ have indicated that postural tremor responds more favorably to botulinum toxin injections than action tremor. These findings collectively support the efficacy of BoNT-A in ameliorating extrapyramidal symptoms. In the present case, the patient’s left upper limb ataxia showed marked improvement following local BoNT-A injections, with 8-point ICARS reduction and 6-point FMA-Upper Extremity Scale improvement, suggesting its potential utility in the treatment of ataxia. However, the precise mechanisms underlying BoNT-A effects on ataxia warrant further investigation.

To date, there are no reported studies on the use of local BoNT-A injections for ataxia treatment, either domestically or internationally. In this case, the patient with thalamic ataxia syndrome exhibited refractory ataxia that was unresponsive to multiple prior treatments. Our approach, involving electromyography- and ultrasound-guided local BoNT-A injections with personalized muscle selection and drug delivery, proved to be both safe and effective. Nevertheless, given the limited number of cases, further case accumulation and systematic efficacy evaluations are necessary to validate these findings.

## 4. Conclusion

The results of this case suggest that BoNT-A may be an effective treatment option for patients with thalamic hemorrhage combined with ataxia. Several limitations should be acknowledged: the single-case design limits generalizability of findings, the short-term follow-up precludes evaluation of sustained treatment effects, and the lack of standardized outcome measures for thalamic ataxia may affect results interpretation. Future studies should further explore the potential of its application in different types of ataxia and evaluate its long-term effects and safety in order to provide a more solid basis for clinical practice.

## Acknowledgments

This case report was written according to the CARE guidelines. The authors appreciate the patients cooperation sincerely.

## Author contributions

**Conceptualization:** Tingting Zhang.

**Formal analysis:** Yike Zhang.

**Writing – original draft:** Jiangbo Xie, Manli Zhao.

**Writing – review & editing:** Peichun Li.
